# (*E*)-3-[(4-Diethyl­amino-2-hydroxy­benzyl­idene)amino]benzonitrile

**DOI:** 10.1107/S1600536809050144

**Published:** 2009-11-28

**Authors:** Jian-Cheng Zhou, Zheng-Yun Zhang, Nai-Xu Li, Chuan-Ming Zhang

**Affiliations:** a College of Chemistry and Chemical Engineering, Southeast University, Nanjing 211189, People’s Republic of China; b Jiangsu Provincial Key Laboratory of Pulp and Paper Science and Technology, Nanjing Forestry University, Nanjing 210037, People’s Republic of China

## Abstract

The mol­ecule of the title compound, C_18_H_19_N_3_O, displays a *trans* configuration with respect to the C=N double bond. There is a strong intra­molecular O—H⋯N hydrogen-bonding inter­action between the hydr­oxy group and imine N atom. The dihedral angle between the aromatic rings is 30.35 (8)°. The crystal packing is stabilized by O—H⋯N links.

## Related literature

For the properties of Schiff bases compounds, see: Zhou *et al.* (2000 ▶); Sriram *et al.* (2006 ▶). For bond-length data, see: Allen *et al.* (1987 ▶).
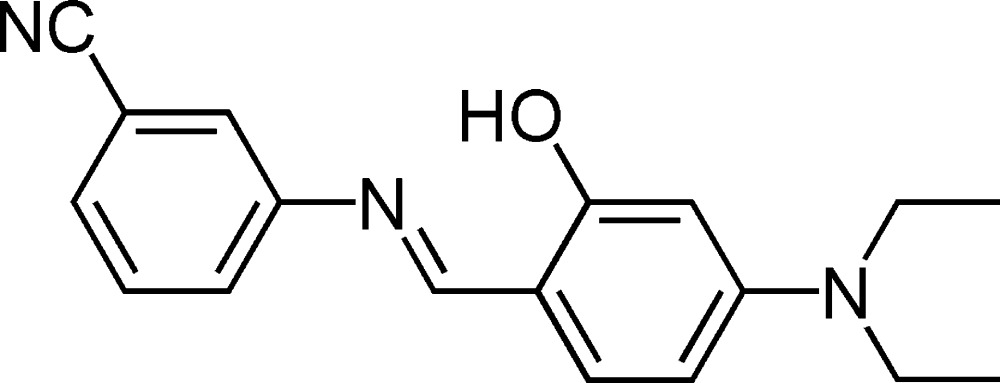


## Experimental

### 

#### Crystal data

C_18_H_19_N_3_O
*M* *_r_* = 293.36
Triclinic, 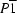

*a* = 8.411 (6) Å
*b* = 8.519 (6) Å
*c* = 12.906 (9) Å
α = 74.17 (4)°
β = 79.00 (4)°
γ = 64.65 (2)°
*V* = 801.1 (9) Å^3^
*Z* = 2
Mo *K*α radiation
μ = 0.08 mm^−1^
*T* = 293 K
0.20 × 0.20 × 0.10 mm

#### Data collection

Rigaku SCXmini diffractometer
Absorption correction: multi-scan (*CrystalClear*; Rigaku, 2005 ▶) *T*_min_ = 0.092, *T*_max_ = 0.182
8686 measured reflections
3620 independent reflections
2592 reflections with *I* > 2σ(*I*)
*R*_int_ = 0.030

#### Refinement

*R*[*F* ^2^ > 2σ(*F* ^2^)] = 0.056
*wR*(*F* ^2^) = 0.159
*S* = 1.09
3620 reflections
203 parameters
1 restraint
H atoms treated by a mixture of independent and constrained refinement
Δρ_max_ = 0.20 e Å^−3^
Δρ_min_ = −0.17 e Å^−3^

### 

Data collection: *CrystalClear* (Rigaku, 2005 ▶); cell refinement: *CrystalClear*; data reduction: *CrystalClear*; program(s) used to solve structure: *SHELXS97* (Sheldrick, 2008 ▶); program(s) used to refine structure: *SHELXL97* (Sheldrick, 2008 ▶); molecular graphics: *SHELXTL* (Sheldrick, 2008 ▶); software used to prepare material for publication: *SHELXL97*.

## Supplementary Material

Crystal structure: contains datablocks I, global. DOI: 10.1107/S1600536809050144/bx2250sup1.cif

Structure factors: contains datablocks I. DOI: 10.1107/S1600536809050144/bx2250Isup2.hkl

Additional supplementary materials:  crystallographic information; 3D view; checkCIF report

## Figures and Tables

**Table 1:** Hydrogen-bond geometry (Å, °)

*D*—H⋯*A*	*D*—H	H⋯*A*	*D*⋯*A*	*D*—H⋯*A*
O1—H1*A*⋯N2	0.98 (3)	1.70 (3)	2.607 (3)	153 (2)
C16—H16*A*⋯O1^i^	0.93	2.60	3.504 (3)	164

Symmetry code: (i) 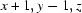.

**Fig. 1.**
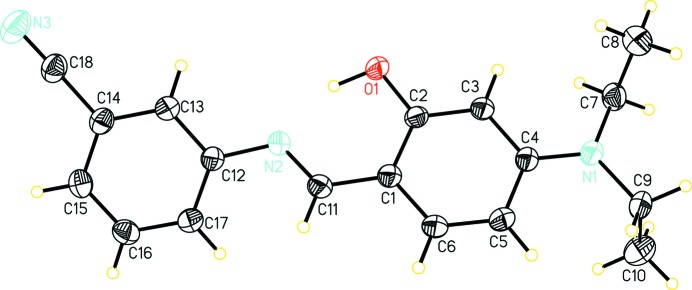
The molecular structure of the title compound, showing the atomic numbering scheme. The displacement ellipsoids are drawn at the 30% probability level.

## supplementary crystallographic information

### Comment

Schiff bases compounds attract great interest in many fields of chemistry and biochemistry, primarily due to significant pharmacological activities, *e.g.* anticancer (Zhou *et al.*, 2000), anti-HIV (Sriram *et al.*, 2006). In addition, Schiff base compounds play important role in the development of coordination chemistry related to magnetism and catalysis. As a continue of my works, we here report the synthesis and crystal structure of the title compound, (I).

The molecular structure of (I) of the title compound is shown in Fig. 1. All the bond lengths and angles in the molecules are in the range of normal values (Allen *et al.*, 1987). The molecule displays a *trans* configuration about the central C11=N2 bond and adopts the phenol-imine tautomeric form, with a strong intramolecular O—H···N hydrogen bonding interaction (Table 1). The dihedral angle between the mean planes of the two aromatic rings is 30.35 (8) ° indicating that the Schiff-base ligand adopts a non-planar conformation. In addition, two methyl groups are positioned to the opposite direction respectively relative to the plane of the adjacent benzene ring. The crystal packing is stabilized by van der Waals interactions.

### Experimental

(*E*)-3-(4-(diethylamino)-2-hydroxybenzylideneamino)benzonitrile was prepared by reflux of a solution mixture containing 4-(diethylamino)-2-hydroxybenzaldehyde (0.996 g, 5 mmol) in ethanol (20 ml) and a solution containing 3-aminobenzonitrile (0.590 g, 5 mmol) in methanol (20 ml). The reaction mixture was stirred for 6 h under reflux, and then cooled to room temperature slowly. The resulting yellow precipitate was filtered off and the crystals of the title compound suitable for X-ray analysis were obtained from acetonitrile solution by slow evaporation

### Refinement

H atoms (for OH) were located in a difference Fourier map and refined isotropically. The remailing H atoms were located geometrically and treated as riding atoms with C—H = 0.93–0.97 Å, and with *U*_iso_(H) = 1.2 *U*_eq_(C) for aromatic H atoms or 1.5 *U*_eq_ (C) for methyl H atoms.

### Crystal data

**Table:**

C_18_H_19_N_3_O	*Z* = 2
*M**_r_* = 293.36	*F*(000) = 312
Triclinic, *P*1	*D*_x_ = 1.216 Mg m^−^^3^
Hall symbol: -P 1	Mo *K*α radiation, λ = 0.71073 Å
*a* = 8.411 (6) Å	Cell parameters from 2426 reflections
*b* = 8.519 (6) Å	θ = 2.3–27.5°
*c* = 12.906 (9) Å	µ = 0.08 mm^−^^1^
α = 74.17 (4)°	*T* = 293 K
β = 79.00 (4)°	Block, yellow
γ = 64.65 (2)°	0.20 × 0.20 × 0.10 mm
*V* = 801.1 (9) Å^3^	

### Data collection

**Table:**

Rigaku SCXmini diffractometer	3620 independent reflections
Radiation source: fine-focus sealed tube	2592 reflections with *I* > 2σ(*I*)
graphite	*R*_int_ = 0.030
Detector resolution: 13.6612 pixels mm^-1^	θ_max_ = 27.5°, θ_min_ = 3.0°
ω scans	*h* = −10→10
Absorption correction: multi-scan (*CrystalClear*; Rigaku, 2005)	*k* = −11→11
*T*_min_ = 0.092, *T*_max_ = 0.182	*l* = −16→16
8686 measured reflections	

### Refinement

**Table:**

Refinement on *F*^2^	Primary atom site location: structure-invariant direct methods
Least-squares matrix: full	Secondary atom site location: difference Fourier map
*R*[*F*^2^ > 2σ(*F*^2^)] = 0.056	Hydrogen site location: inferred from neighbouring sites
*wR*(*F*^2^) = 0.159	H atoms treated by a mixture of independent and constrained refinement
*S* = 1.09	*w* = 1/[σ^2^(*F*_o_^2^) + (0.0841*P*)^2^] where *P* = (*F*_o_^2^ + 2*F*_c_^2^)/3
3620 reflections	(Δ/σ)_max_ < 0.001
203 parameters	Δρ_max_ = 0.20 e Å^−^^3^
1 restraint	Δρ_min_ = −0.16 e Å^−^^3^

### Special details

**Table:**

Geometry. All e.s.d.'s (except the e.s.d. in the dihedral angle between two l.s. planes) are estimated using the full covariance matrix. The cell e.s.d.'s are taken into account individually in the estimation of e.s.d.'s in distances, angles and torsion angles; correlations between e.s.d.'s in cell parameters are only used when they are defined by crystal symmetry. An approximate (isotropic) treatment of cell e.s.d.'s is used for estimating e.s.d.'s involving l.s. planes.
Refinement. Refinement of *F*^2^ against ALL reflections. The weighted *R*-factor *wR* and goodness of fit *S* are based on *F*^2^, conventional *R*-factors *R* are based on *F*, with *F* set to zero for negative *F*^2^. The threshold expression of *F*^2^ > σ(*F*^2^) is used only for calculating *R*-factors(gt) *etc*. and is not relevant to the choice of reflections for refinement. *R*-factors based on *F*^2^ are statistically about twice as large as those based on *F*, and *R*- factors based on ALL data will be even larger.

### Fractional atomic coordinates and isotropic or equivalent isotropic displacement parameters (Å^2^)

**Table:**

	*x*	*y*	*z*	*U*_iso_*/*U*_eq_	
C3	0.2014 (2)	1.0630 (2)	0.37779 (12)	0.0428 (4)	
H3A	0.0814	1.1144	0.3988	0.051*	
O1	0.24337 (15)	0.87525 (17)	0.55059 (9)	0.0544 (3)	
N2	0.56035 (17)	0.63888 (18)	0.59125 (10)	0.0456 (3)	
C1	0.4976 (2)	0.8526 (2)	0.42381 (12)	0.0417 (4)	
C4	0.2656 (2)	1.1212 (2)	0.27203 (12)	0.0415 (4)	
C13	0.6447 (2)	0.4627 (2)	0.76732 (12)	0.0455 (4)	
H13A	0.5454	0.5452	0.7974	0.055*	
N1	0.15321 (18)	1.24256 (19)	0.19656 (10)	0.0503 (4)	
C2	0.31405 (19)	0.9308 (2)	0.45073 (11)	0.0396 (4)	
C11	0.6144 (2)	0.7100 (2)	0.49704 (12)	0.0458 (4)	
H11A	0.7348	0.6662	0.4761	0.055*	
C6	0.5599 (2)	0.9187 (2)	0.31953 (12)	0.0485 (4)	
H6A	0.6808	0.8729	0.3003	0.058*	
C12	0.6806 (2)	0.4883 (2)	0.65573 (12)	0.0424 (4)	
C9	0.2167 (2)	1.3062 (2)	0.08716 (13)	0.0547 (4)	
H9A	0.3286	1.3124	0.0891	0.066*	
H9B	0.1333	1.4255	0.0594	0.066*	
C17	0.8278 (2)	0.3586 (2)	0.61325 (13)	0.0493 (4)	
H17A	0.8512	0.3716	0.5387	0.059*	
C5	0.4508 (2)	1.0469 (2)	0.24531 (13)	0.0490 (4)	
H5A	0.4979	1.0858	0.1769	0.059*	
C18	0.7203 (2)	0.2892 (2)	0.95013 (14)	0.0548 (4)	
C14	0.7571 (2)	0.3138 (2)	0.83397 (12)	0.0465 (4)	
C15	0.9066 (2)	0.1876 (2)	0.79080 (14)	0.0511 (4)	
H15A	0.9828	0.0895	0.8358	0.061*	
C7	−0.0392 (2)	1.3053 (2)	0.21951 (13)	0.0533 (4)	
H7A	−0.0677	1.2098	0.2678	0.064*	
H7B	−0.0928	1.3357	0.1527	0.064*	
C16	0.9387 (2)	0.2118 (2)	0.68022 (14)	0.0524 (4)	
H16A	1.0366	0.1278	0.6502	0.063*	
N3	0.6938 (2)	0.2678 (3)	1.04170 (13)	0.0772 (5)	
C10	0.2415 (3)	1.1884 (3)	0.01095 (15)	0.0798 (7)	
H10A	0.2833	1.2364	−0.0598	0.120*	
H10B	0.1307	1.1837	0.0074	0.120*	
H10C	0.3261	1.0707	0.0370	0.120*	
C8	−0.1160 (3)	1.4645 (3)	0.27007 (17)	0.0716 (6)	
H8A	−0.2415	1.4999	0.2842	0.107*	
H8B	−0.0912	1.5606	0.2217	0.107*	
H8C	−0.0644	1.4348	0.3367	0.107*	
H1A	0.342 (3)	0.773 (4)	0.5846 (19)	0.109 (8)*	

### Atomic displacement parameters (Å^2^)

**Table:**

	*U*^11^	*U*^22^	*U*^33^	*U*^12^	*U*^13^	*U*^23^
C3	0.0344 (8)	0.0488 (9)	0.0402 (8)	−0.0142 (7)	−0.0010 (6)	−0.0073 (7)
O1	0.0396 (6)	0.0676 (8)	0.0374 (6)	−0.0133 (6)	0.0027 (5)	−0.0001 (5)
N2	0.0419 (8)	0.0490 (8)	0.0392 (7)	−0.0114 (6)	−0.0061 (6)	−0.0083 (6)
C1	0.0373 (8)	0.0466 (9)	0.0390 (8)	−0.0149 (7)	−0.0021 (6)	−0.0094 (7)
C4	0.0416 (9)	0.0429 (9)	0.0383 (8)	−0.0158 (7)	−0.0052 (6)	−0.0064 (6)
C13	0.0448 (9)	0.0481 (9)	0.0412 (8)	−0.0157 (7)	−0.0031 (7)	−0.0110 (7)
N1	0.0456 (7)	0.0564 (9)	0.0376 (7)	−0.0148 (6)	−0.0043 (6)	−0.0010 (6)
C2	0.0368 (8)	0.0470 (9)	0.0343 (7)	−0.0168 (7)	0.0004 (6)	−0.0098 (6)
C11	0.0369 (8)	0.0526 (10)	0.0441 (9)	−0.0137 (7)	−0.0029 (6)	−0.0117 (7)
C6	0.0345 (8)	0.0561 (10)	0.0461 (9)	−0.0145 (7)	0.0030 (7)	−0.0076 (7)
C12	0.0408 (8)	0.0466 (9)	0.0394 (8)	−0.0157 (7)	−0.0053 (6)	−0.0099 (7)
C9	0.0568 (11)	0.0526 (10)	0.0429 (9)	−0.0192 (8)	−0.0063 (7)	0.0051 (7)
C17	0.0516 (10)	0.0518 (10)	0.0418 (9)	−0.0161 (8)	−0.0019 (7)	−0.0146 (7)
C5	0.0442 (9)	0.0556 (10)	0.0408 (8)	−0.0203 (8)	0.0046 (7)	−0.0053 (7)
C18	0.0590 (11)	0.0590 (11)	0.0458 (10)	−0.0276 (9)	−0.0059 (8)	−0.0023 (8)
C14	0.0513 (9)	0.0497 (9)	0.0415 (8)	−0.0251 (8)	−0.0050 (7)	−0.0056 (7)
C15	0.0520 (10)	0.0435 (9)	0.0543 (10)	−0.0166 (8)	−0.0121 (8)	−0.0039 (7)
C7	0.0477 (8)	0.0562 (11)	0.0507 (10)	−0.0176 (8)	−0.0121 (7)	−0.0029 (8)
C16	0.0491 (10)	0.0446 (10)	0.0585 (11)	−0.0124 (8)	−0.0015 (8)	−0.0159 (8)
N3	0.0913 (13)	0.0963 (14)	0.0452 (9)	−0.0474 (11)	−0.0026 (8)	−0.0027 (8)
C10	0.0944 (17)	0.0927 (16)	0.0488 (11)	−0.0326 (13)	−0.0029 (10)	−0.0197 (11)
C8	0.0696 (13)	0.0594 (12)	0.0721 (13)	−0.0158 (10)	−0.0011 (10)	−0.0126 (10)

### Geometric parameters (Å, °)

**Table:**

C3—C2	1.379 (2)	C9—C10	1.516 (3)
C3—C4	1.407 (2)	C9—H9A	0.9700
C3—H3A	0.9300	C9—H9B	0.9700
O1—C2	1.3616 (19)	C17—C16	1.379 (2)
O1—H1A	0.98 (3)	C17—H17A	0.9300
N2—C11	1.294 (2)	C5—H5A	0.9300
N2—C12	1.410 (2)	C18—N3	1.139 (2)
C1—C6	1.404 (2)	C18—C14	1.447 (2)
C1—C2	1.410 (2)	C14—C15	1.396 (2)
C1—C11	1.428 (2)	C15—C16	1.376 (2)
C4—N1	1.370 (2)	C15—H15A	0.9300
C4—C5	1.420 (2)	C7—C8	1.502 (3)
C13—C14	1.390 (2)	C7—H7A	0.9700
C13—C12	1.390 (2)	C7—H7B	0.9700
C13—H13A	0.9300	C16—H16A	0.9300
N1—C9	1.454 (2)	C10—H10A	0.9600
N1—C7	1.468 (2)	C10—H10B	0.9600
C11—H11A	0.9300	C10—H10C	0.9600
C6—C5	1.363 (2)	C8—H8A	0.9600
C6—H6A	0.9300	C8—H8B	0.9600
C12—C17	1.398 (2)	C8—H8C	0.9600
			
C2—C3—C4	120.82 (15)	C16—C17—C12	120.98 (15)
C2—C3—H3A	119.6	C16—C17—H17A	119.5
C4—C3—H3A	119.6	C12—C17—H17A	119.5
C2—O1—H1A	104.4 (14)	C6—C5—C4	120.24 (15)
C11—N2—C12	120.13 (14)	C6—C5—H5A	119.9
C6—C1—C2	116.50 (14)	C4—C5—H5A	119.9
C6—C1—C11	121.36 (15)	N3—C18—C14	179.0 (2)
C2—C1—C11	122.11 (14)	C13—C14—C15	121.09 (15)
N1—C4—C3	121.23 (15)	C13—C14—C18	119.75 (16)
N1—C4—C5	121.00 (14)	C15—C14—C18	119.16 (16)
C3—C4—C5	117.75 (15)	C16—C15—C14	118.49 (16)
C14—C13—C12	120.03 (15)	C16—C15—H15A	120.8
C14—C13—H13A	120.0	C14—C15—H15A	120.8
C12—C13—H13A	120.0	N1—C7—C8	112.48 (16)
C4—N1—C9	122.17 (15)	N1—C7—H7A	109.1
C4—N1—C7	121.48 (14)	C8—C7—H7A	109.1
C9—N1—C7	116.19 (13)	N1—C7—H7B	109.1
O1—C2—C3	118.17 (14)	C8—C7—H7B	109.1
O1—C2—C1	120.13 (14)	H7A—C7—H7B	107.8
C3—C2—C1	121.70 (14)	C15—C16—C17	120.95 (16)
N2—C11—C1	123.02 (15)	C15—C16—H16A	119.5
N2—C11—H11A	118.5	C17—C16—H16A	119.5
C1—C11—H11A	118.5	C9—C10—H10A	109.5
C5—C6—C1	122.87 (15)	C9—C10—H10B	109.5
C5—C6—H6A	118.6	H10A—C10—H10B	109.5
C1—C6—H6A	118.6	C9—C10—H10C	109.5
C13—C12—C17	118.42 (15)	H10A—C10—H10C	109.5
C13—C12—N2	118.03 (14)	H10B—C10—H10C	109.5
C17—C12—N2	123.44 (14)	C7—C8—H8A	109.5
N1—C9—C10	112.94 (17)	C7—C8—H8B	109.5
N1—C9—H9A	109.0	H8A—C8—H8B	109.5
C10—C9—H9A	109.0	C7—C8—H8C	109.5
N1—C9—H9B	109.0	H8A—C8—H8C	109.5
C10—C9—H9B	109.0	H8B—C8—H8C	109.5
H9A—C9—H9B	107.8		

### Hydrogen-bond geometry (Å, °)

**Table:**

*D*—H···*A*	*D*—H	H···*A*	*D*···*A*	*D*—H···*A*
O1—H1A···N2	0.98 (3)	1.70 (3)	2.607 (3)	153 (2)
C16—H16A···O1^i^	0.93	2.60	3.504 (3)	164

Symmetry codes: (i) *x*+1, *y*−1, *z*.
